# Supplementary material for: Multi‐dataset identification of innovative feature genes and molecular mechanisms in keratoconus
Source: J Cell Mol Med. 2024 Sep 19;28(18):e70079. doi: 10.1111/jcmm.70079 (PMC11412914; doi:10.1111/jcmm.70079)
Supplement: Supplementary file 2 — Material S2. [file JCMM-28-e70079-s002.zip › 7.GSEA analysis/6.SNORA74B/KEGG_GLYCINE_SERINE_AND_THREONINE_METABOLISM.html]

Details for gene set KEGG\_GLYCINE\_SERINE\_AND\_THREONINE\_METABOLISM[GSEA]

|  || Dataset | SNORA74B.group.cls#h\_versus\_l |
| Phenotype | group.cls#h\_versus\_l |
| Upregulated in class | h |
| GeneSet | KEGG\_GLYCINE\_SERINE\_AND\_THREONINE\_METABOLISM |
| Enrichment Score (ES) | 0.2664013 |
| Normalized Enrichment Score (NES) | 0.9081486 |
| Nominal p-value | 0.66528064 |
| FDR q-value | 1.0 |
| FWER p-Value | 1.0 |
Table: GSEA Results Summary

  

Fig 1: Enrichment plot: KEGG\_GLYCINE\_SERINE\_AND\_THREONINE\_METABOLISM      
 Profile of the Running ES Score & Positions of GeneSet Members on the Rank Ordered List

  

| SYMBOL | TITLE | RANK IN GENE LIST | RANK METRIC SCORE | RUNNING ES | CORE ENRICHMENT || 1 | AOC2 | na | 747 | 0.534 | 0.0752 | Yes |
| 2 | MAOB | na | 1824 | 0.387 | 0.1144 | Yes |
| 3 | DMGDH | na | 3137 | 0.294 | 0.1298 | Yes |
| 4 | ALAS2 | na | 3264 | 0.288 | 0.1783 | Yes |
| 5 | GLYCTK | na | 3587 | 0.274 | 0.2188 | Yes |
| 6 | AGXT2 | na | 3647 | 0.272 | 0.2664 | Yes |
| 7 | BHMT | na | 6730 | 0.170 | 0.2084 | No |
| 8 | GAMT | na | 8668 | 0.121 | 0.1744 | No |
| 9 | DLD | na | 12649 | 0.051 | 0.0690 | No |
| 10 | SHMT1 | na | 13090 | 0.046 | 0.0646 | No |
| 11 | GLDC | na | 13228 | 0.044 | 0.0687 | No |
| 12 | SRR | na | 14311 | 0.032 | 0.0434 | No |
| 13 | GNMT | na | 15038 | 0.024 | 0.0269 | No |
| 14 | MAOA | na | 16871 | 0.006 | -0.0249 | No |
| 15 | SHMT2 | na | 20121 | -0.030 | -0.1131 | No |
| 16 | PIPOX | na | 21698 | -0.048 | -0.1499 | No |
| 17 | DAO | na | 24207 | -0.084 | -0.2069 | No |
| 18 | GCAT | na | 24501 | -0.089 | -0.1992 | No |
| 19 | GATM | na | 24879 | -0.096 | -0.1926 | No |
| 20 | PSAT1 | na | 25464 | -0.107 | -0.1901 | No |
| 21 | ALAS1 | na | 25821 | -0.113 | -0.1799 | No |
| 22 | AMT | na | 26650 | -0.131 | -0.1800 | No |
| 23 | SDS | na | 27794 | -0.159 | -0.1840 | No |
| 24 | PHGDH | na | 28234 | -0.171 | -0.1656 | No |
| 25 | SARDH | na | 28596 | -0.182 | -0.1430 | No |
| 26 | PSPH | na | 29205 | -0.201 | -0.1242 | No |
| 27 | AGXT | na | 29339 | -0.204 | -0.0910 | No |
| 28 | CBS | na | 31069 | -0.272 | -0.0916 | No |
| 29 | CTH | na | 31622 | -0.302 | -0.0528 | No |
| 30 | AOC3 | na | 32580 | -0.359 | -0.0153 | No |
| 31 | CHDH | na | 33316 | -0.426 | 0.0407 | No |
Table: GSEA details [plain text format]

  

Fig 2: KEGG\_GLYCINE\_SERINE\_AND\_THREONINE\_METABOLISM      
 Blue-Pink O' Gram in the Space of the Analyzed GeneSet

  

Fig 3: KEGG\_GLYCINE\_SERINE\_AND\_THREONINE\_METABOLISM: Random ES distribution      
 Gene set null distribution of ES for **KEGG\_GLYCINE\_SERINE\_AND\_THREONINE\_METABOLISM**
